# The Dutch version of the Child Posttraumatic Cognitions Inventory: validation in a clinical sample and a school sample

**DOI:** 10.3402/ejpt.v6.26362

**Published:** 2015-02-23

**Authors:** Julia Diehle, Carlijn de Roos, Richard Meiser-Stedman, Frits Boer, Ramón J. L. Lindauer

**Affiliations:** 1 Department of Child and Adolescent Psychiatry, Academic Medical Centre, Amsterdam, The Netherlands; 2 Psychotrauma Center for Children and Adolescents, MHI Rivierduinen, Leiden, The Netherlands; 3 Medical Research Council Cognition & Brain Sciences Unit, Cambridge, United Kingdom; 4 Academic Center for Child and Adolescent Psychiatry de Bascule, Amsterdam, The Netherlands

**Keywords:** posttraumatic stress disorder, children, cognitions, reliability, validity

## Abstract

**Background:**

With the inclusion of trauma-related cognitions in the DSM-5 criteria for posttraumatic stress disorder (PTSD), the assessment of these cognitions has become essential. Therefore, valid tools for the assessment of these cognitions are warranted.

**Objective:**

The current study aimed at validating the Dutch version of the Child Posttraumatic Cognitions Inventory (CPTCI).

**Method:**

We included children aged 8–19 years in our study and assessed the factor structure, reliability and validity of the CPTCI in a clinical sample (*n*=184) and a school sample (*n*=318).

**Results:**

Our results supported the two-factor structure of the CPTCI and showed good internal consistency for the total scale and the two subscales. We found significant positive correlations between the CPTCI and measures of PTSD, depression, and anxiety disorder. The CPTCI correlated negatively with a measure of quality of life. Furthermore, we found significantly higher scores in the clinical sample than in the school sample. For children who received treatment, we found that a decrease in CPTCI scores was accompanied by a decrease in posttraumatic stress symptoms and comorbid problems indicating that the CPTCI is able to detect treatment effects.

**Conclusion:**

Overall, our results suggest that the Dutch CPTCI is a reliable and valid instrument.

In the past years, researchers have repeatedly demonstrated that people who suffer from posttraumatic stress disorder (PTSD) according to the criteria of the 4th edition of the Diagnostic and Statistical Manual of Mental Disorders (text rev. [DSM-IV-TR], American Psychiatric Association [APA], 2000) also suffer from trauma-related cognitions (e.g., Agar, Kennedy, & King, 2006; Dunmore, Clark, & Ehlers, 1999). As a consequence, trauma-related cognitions have become part of the criteria for PTSD in the DSM-5 (APA, 2013). This change is reflected in the newly included cluster “negative alterations in cognitions and mood,” specifically in items D2: persistent (and often distorted) negative beliefs and expectations about oneself or the world (e.g., “I am bad,” “The world is completely dangerous”), and D3: persistent distorted blame of self or others for causing the traumatic event or for resulting consequences (APA, 2013). By including trauma-related cognitions in the criteria for PTSD, the assessment of these cognitions has become essential. However, until now, questionnaires and interviews measuring PTSD lack the component of trauma-related cognitions. Therefore, reliable and valid assessment tools are needed that measure trauma-related cognitions.

For adults, there are currently several questionnaires that measure trauma-related cognitions. One of these is the Posttraumatic Cognitions Inventory (PTCI), which has been implemented for the assessment of trauma-related cognitions over the past years. Foa, Ehlers, Clark, Tolin, and Orsillo (1999) developed this 33-items self-report questionnaire, which has shown good psychometric properties. The three subscales measure negative beliefs about the self, negative beliefs about the world and self-blame. The PTCI has often been used in research and has been validated in English-speaking populations and in Germany, the Netherlands, the State of Israel and Taiwan, too (Beck et al., 2004; Daie-Gabai, Aderka, Allon-Schindel, Foa, & Gilboa-Schechtman, 2011; Müller et al., 2010; Su & Chen, 2008; Van Emmerik, Schoorl, Emmelkamp, & Kamphuis, 2006). These validation studies replicated the factor structure of the PTCI and demonstrated that it is a valid and reliable instrument. Results also indicate that participant characteristics like gender, experienced trauma type and cultural background can substantially influence the scores on and also the psychometric properties of the PTCI.

An even more influential factor might be age. There is general agreement that adult diagnostic instruments cannot be used in children without adaptation. Therefore, Meiser-Stedman, Smith, et al. (2009) developed the child version of the PTCI. After linguistic changes and item reductions, the Child Posttraumatic Cognitions Inventory (CPTCI) consists of 25 age appropriate items. In contrast to the adult version, the CPTCI consists only of two subscales. Thirteen items compose the permanent and disturbing change subscale (CPTCI-PC). These items focus on the negative effect the frightening event had on the child and his/her perception of the future in the light of the frightening event. The fragile person in a scary world subscale (CPTCI-SW) comprises the remaining 12 items. These items inquire about the child's own sense of weakness and the perception of the world and other people as threatening. As a result of the item reduction, the five items that were part of the self-blame subscale in the adult version are not included in the CPTCI. The original validation study of the CPTCI (Meiser-Stedman, Smith, et al., 2009) took place in three samples: UK secondary school pupils; UK children who had been exposed to a motor vehicle accident or assault 3 months earlier; and Australian children who had been admitted to hospital after an injury. Factor loadings differed in the three samples and on the CPTCI-SW subscale some items showed only small factor loadings (<0.30). Still, the CPTCI proved to be reliable with good internal consistency for the subscales and the total scale and good test–retest reliability. Furthermore, the questionnaire correlated strongly with measures of PTSD and depression and was able to discriminate between children with and without PTSD and between children with and without acute stress disorder (ASD, Meiser-Stedman, Smith, et al., 2009). In further research, the CPTCI has mostly been put into practice to investigate its predictive effects for ASD and PTSD. These studies showed that scores on the CPTCI indeed predict PTSD and ASD (Bryant, Salmon, Sinclair, & Davidson, 2007; Meiser-Stedman, Dalgleish, Glucksman, Yule, & Smith, 2009; Salmon, Sinclair, & Bryant, 2007). In the field of treatment outcome, Smith et al. (2007) found a strong significant correlation between changes in PTSD symptoms and changes on the CPTCI, indicating that the CPTCI is able to measure treatment effects. This was supported by Nixon, Sterk and Pearce (2012) who found a significant reduction on the CPTCI from pre- to posttrauma therapy.

Different studies have demonstrated that the CPTCI showed good internal consistency in children who had experienced a single traumatic event and also in children who had been exposed to psychological maltreatment (Leeson & Nixon, 2011; Salmond et al., 2011). However, a more thorough investigation of the factor structure and the psychometric properties of the CPTCI is lacking. It is important to replicate the findings by Meiser-Stedman, Smith, et al. (2009) and to investigate the psychometric properties of the CPTCI in a more heterogeneous sample, i.e., in children who were exposed to different kinds of traumatic events and who were exposed not only to single-event trauma but also to multiple-event trauma. Furthermore, although the CPTCI has been translated into more than 10 languages and translated versions have also been used in scientific research (e.g., Palosaari, Punamäki, Diab, & Quota, 2013), a cross-cultural validation of the instrument is still lacking. Hence, it is yet unknown if the translation of the questionnaire has as good psychometric properties as the original one and can be used without major adaptations. Therefore, the goal of the current study was to validate the Dutch CPTCI. We want to re-evaluate the psychometric properties of the CPTCI in a more heterogeneous sample and add a cross-cultural validation to the scientific literature.

## Method

### Sample

We collected data from 502 children and adolescents aged 8–19 years for our study. Participants were recruited at two centers for child and adolescent trauma (de Bascule, academic center for child and adolescent psychiatry in Amsterdam; and the Mental Health Institution Rivierduinen, child and adolescent department in Leiden), and at different primary and secondary schools in the region of Amsterdam. The school sample consisted of 318 children aged 8–17 years (*M*=13.34; *SD*=2.62). One hundred and fifty-five (49%) of them were boys. Children were instructed to bear in mind the most frightening event they had experienced in their life when completing the CPTCI. Thirty-one percent reported that the death of a loved one was the most frightening experience they had experienced so far. Accidents, divorce/fights between parents and being teased were also frequently reported (12, 10, and 10%, respectively). Fifteen percent of all school children reported an event that could be classified as traumatic. Children who were seeking treatment at one of the centers for child and adolescent trauma (clinical sample) filled out the CPTCI in relation to the core traumatic event for which they sought treatment. This sample comprised 184 children aged 8–19 years (*M*=13.39; *SD*=3.23). Seventy-five (41%) of them were boys. Most frequently reported events were sexual abuse (23%) and traumatic loss (11%). Thirty-four (19%) children had been exposed to multiple-event trauma (meaning that they had repeatedly been exposed to traumatic events in the past), whereas 149 (81%) reported at referral that they had been exposed to a single-event trauma.

### Procedure

In the clinical sample, parents and children older than 11 years signed an informed consent form. The CPTCI and Revised Child Anxiety and Depression Scale (RCADS, Chorpita, Yim, Moffitt, Umemoto, & Francis, 2000) were administered as part of the standard diagnostic procedure before and after trauma-focused treatment (treatment was either Trauma-Focused Cognitive Behavioral Therapy or Eye Movement Desensitization and Reprocessing). In Amsterdam the Clinician-Administered PTSD Scale for Children and Adolescents (CAPS-CA, Nader et al., 1996) was also part of this procedure. Children in the school sample and their parents received either a letter with information about the study or were informed via the newsletter of the school. Parents of children younger than 12 years gave active informed consent for the participation of their child. Parents and children 12 years and older were asked for passive informed consent. School children filled out the CPTCI and the KIDSCREEN-10 (Ravens-Sieberer et al., 2005; The KIDSCREEN Group Europe, 2006) at school during regular school hours.

### Measures

#### CPTCI (Meiser-Stedman, Smith, et al., 2009)

The CPTCI is a self-report questionnaire that investigates trauma-related cognitions in children and adolescents. The original English version has been validated in children aged 6–18 years. The 25 items of the questionnaire can be rated on a four-point Likert scale ranging from 1 “Don't agree at all” to 4 “Agree a lot.” The two subscales as well as the total scale have shown good internal consistency (Cronbach's *α* 0.86–0.93). Strong correlations with measures of PTSD [Children's Revised Impact of Event Scale (Perrin, Meiser-Stedman, & Smith, 2005) and Child Posttraumatic Stress Scale (Foa, Johnson, Feeny, & Treadwell, 2001), *r*>0.5] and depression [Depression Self-Rating Scale for children (Birleson, 1981), *r*>0.6] indicated good convergent validity. The CPTCI was also able to discriminate between children with and without ASD and with and without PTSD. For the present study, the English version of the CPTCI was translated into Dutch by a group of native Dutch and Dutch-speaking child and adolescent psychologists and psychiatrists and back-translated into English by a native English speaker. The back-translation was sent to one of the original authors (Meiser-Stedman) who approved of this version.

#### CAPS-CA (Nader et al., 1996)

This semistructured clinical interview was designed to investigate PTSD according to the DSM-IV-TR standards. It is known as the gold standard diagnostic tool for PTSD in children aged 8–18 years. The interviewer can score the frequency and the intensity of each symptom on a five-point Likert scale. The severity score for each of the 17 items is calculated by adding up the frequency and intensity score. The total PTSD severity score is the sum of the severity scores for all 17 items (in the range 0–136). In the current study, the CAPS-CA was administered by trained psychologists. Inter-rater reliability was excellent with an intraclass correlation coefficient for the total scale of 0.99 and a κ statistic of 0.75 for agreement on PTSD diagnosis. The Dutch CAPS-CA has shown good internal reliability for the three subscales and the total scale with Cronbach's *α* ranging between 0.77 and 0.83 (Diehle, De Roos, Boer, & Lindauer, 2013).

#### RCADS (Chorpita et al., 2000)

The RCADS is a 47-item self-report questionnaire with six subscales: social phobia, panic disorder, generalized anxiety disorder, major depressive disorder, separation anxiety disorder, and obsessive compulsive disorder. Combined scores of all items result in the total internalizing score. Items can be rated on a four-point Likert scale. Cronbach's *α* for the six subscales indicated good internal consistency (*α*=0.71–0.85; Chorpita et al., 2000). In the current study we found *α*'s ranging between 0.73 and 0.89.

#### KIDSCREEN-10 (Ravens-Sieberer et al., 2005; The KIDSCREEN Group Europe, 2006)

The KIDSCREEN-10 is short questionnaire about health-related quality of life. It consists of 10 items that can be scored on a five-point Likert scale. The validation of the questionnaire in a multinational research project showed that it has adequate psychometric properties with Cronbach's *α* of 0.82 (Ravens-Sieberer et al., 2010). We found *α=*0.84 in the current study.

### Statistical analysis

We took the following steps to answer our research question: first we performed separate confirmatory factor analyses (CFAs) for the clinical and the school sample using R 3.0.1 and the lavaan package for CFA. We tested the original two-factor structure of the CPTCI as specified by Meiser-Stedman, Smith, et al. (2009) and assigned 13 items to the CPTCI-PC subscale and 12 items to the CPTCI-SW subscale (see Table 1). Items were constrained to load only on the designated factor. We also tested a one-factor model (i.e., all 25 items of the CPTCI in a single factor) so as to provide a more parsimonious comparator model to the two-factor model. If the two-factor model was a superior fit to the data than the one-factor model, this would support the continued use of the CPTCI's two subscales, “permanent and disturbing change” and “fragile person in a scary world,” rather than just a total score. Since scores were non-normally distributed, we adopted the standard maximum likelihood estimation with robust standard errors and a Satorra-Bentler scaled test statistic (Rosseel, 2012). A good fit of the model is achieved if the comparative fit index (CFI) is larger than 0.95 and if the root-mean square approximation (RMSEA) is lower than 0.06 (Hu & Bentler, 1999). Second, we examined the internal consistency by calculating Cronbach's *α* for the total and the two subscales. In a third step, we calculated Pearson correlation coefficients between the CPTCI and measures of PTSD, anxiety, and depression, and quality of life to inspect convergent validity. In a subanalysis, we investigated the correlations between the CPTCI and the CAPS-CA or RCADS separately for children who experienced single-event trauma and children who experienced multiple-event trauma. Fourth, we investigated if the CPTCI was able to discriminate between children in the school sample and children in the clinical sample by means of an independent samples *t*-test. Fifth to find out whether the CPTCI is able to detect treatment effects, we examined the correlation between pre- to posttreatment change scores on the CPTCI and change scores on the CAPS-CA and change scores on the RCADS. Sixth we additionally calculated an independent samples *t*-test for the comparison of boys and girls and used analysis of variance with post hoc Bonferroni correction for the comparison of different age groups. Apart from the CFA, all analyses were performed using SPSS version 21. For the calculations of correlations and comparisons, we allowed 20% missing values per subscale for each questionnaire. Missing values were replaced by the individual mean of the valid items of the subscale.

**Table 1:** Factor loadings of the CPTCI items by sample

Item	CPTCI-PC		CPTCI-SW	
	
	Clinic	School	Clinic	School
4. My reactions since the frightening event mean I have changed for the worse.	0.57	0.68		
6. My reactions since the frightening event mean something is seriously wrong with me.	0.64	0.58		
8. Not being able to get over all my fears means that I am a failure.	0.66	0.56		
13. My reactions since the frightening event mean I will never get over it.	0.67	0.70		
14. I used to be a happy person but now I am always sad.	0.63	0.59		
16. I will never be able to have normal feelings again.	0.71	0.62		
17. I'm scared that I'll get so angry that I'll break something or hurt someone.	0.56	0.44		
19. My life has been destroyed by the frightening event.	0.76	0.70		
20. I feel like I am a different person since the frightening event.	0.74	0.67		
21. My reactions since the frightening event show that I must be going crazy.	0.70	0.66		
22. Nothing good can happen to me anymore.	0.62	0.56		
23. Something terrible will happen if I do not try to control my thoughts about the frightening event.	0.51	0.55		
24. The frightening event has changed me forever.	0.67	0.54		
1. Anyone could hurt me.			0.42	0.44
2. Everyone lets me down.			0.66	0.55
3. I am a coward.			0.60	0.43
5. I don't trust people.			0.57	0.46
7. I am no good.			0.75	0.54
9. Small things upset me.			0.58	0.57
10. I can't cope when things get tough.			0.47	0.46
11. I can't stop bad things from happening to me.			0.50	0.59
12. I have to watch out for danger all the time.			0.42	0.56
15. Bad things always happen.			0.74	0.62
18. Life is not fair.			0.68	0.32
25. I have to be really careful because something bad could happen.			0.50	0.61

CPTCI=Child Posttraumatic Cognitions Inventory; CPTCI-PC=permanent and disturbing change subscale of the CPTCI; CPTCI-SW= fragile person in a scary world subscale of the CPTCI; clinic=clinical sample; school=school sample.

## Results

### Confirmatory factor analysis

The one-factor model provided for the clinical as well as for the school sample, a mediocre fit of the data; clinical sample: S-B *χ* ^2^ (275)=493.41, *p*<0.001, CFI=0.85, and RMSEA=0.07, 95% CI [0.06, 0.08]; school sample: S-B *χ* ^2^ (275)=546.65, *p<*0.001, CFI=0.83, and RMSEA=0.06, 95% CI [0.05, 0.06]. The specified two-factor model provided a slightly better fit of the data, for both, the clinical sample: S-B *χ* ^2^ (274)=481.97, *p*<0.001, CFI=0.86, and RMSEA=0.07, 95% CI [0.06, 0.08]; and the school sample: S-B *χ* ^2^ (274)=507.33, *p*<0.001, CFI=0.86, and RMSEA=0.05, 95% CI [0.05, 0.06]. For the two-factor model, factor loadings were all larger than 0.40 except for item 18 in the school sample. Here the factor loading was only 0.32 (see Table 1).

### Internal consistency

Cronbach's *α*'s for the CPTCI-PC and CPTCI-SW subscales were comparable in the clinical and the school sample with 0.90 and 0.85 in the clinical and 0.87 and 0.80 in the school sample. Like Meiser-Stedman, Smith, et al. (2009), we also investigated Cronbach's *α* for different age groups (8–11, 12–15, and 16–19). We found the smallest *α* of 0.68 for the CPTCI-SW subscale in the youngest age group. All other *α*'s were >0.84. We also computed in the clinical sample Cronbach's *α* for children who were exposed to a single-event trauma and children who were exposed multiple-event trauma. Cronbach's *α*'s were slightly higher in the latter group with 0.92 and 0.87 vs. 0.89 and 0.84 in the single trauma group.

### Validity

As displayed in Table 2, the CPTCI showed strong positive correlations with the CAPS-CA total and subscales and with most subscales of the RCADS. As expected for the correlations between the CPTCI and the RCADS, we found the strongest correlation with the subscale “major depressive disorder.” Given the strong correlations between the RCADS and the CPTCI, we calculated partial correlations for the CPTCI and CAPS-CA. We controlled for the RCADS total internalizing score to ensure that the correlation between the CPTCI and the CAPS-CA was not just an artifact of the relationship between the trauma-related cognitions and anxiety and depression symptoms. Our results showed that the CPTCI-PC, CPTCI-SW, and CPTCI total scale continued to significantly correlate with the CAPS-CA total severity score: *r*=0.34, 0.32, and 0.37 (all *p* values<0.01). When controlling for either CPTCI subscale, the partial correlation between the CAPS-CA total severity score and the CPTCI-PC was significant (*r*=0.25, *p*<0.05), whereas partial correlations between the CAPS-CA total severity score and the CPTCI-SW subscale just fell short of significance (*r*=0.22, *p*=0.053). We also investigated the correlations between the CPTCI and the CAPS-CA total severity score and the RCADS total internalizing score separately for children who were exposed to single-event trauma and children who were exposed to multiple-event trauma. We found for both groups strong, significant correlations between the CPTCI and the CAPS-CA which were slightly larger in the single-event trauma group (*n*=48) ranging between *r*=0.69 and *r*=0.72. (all *p* values<0.001) Correlations in the multiple-event trauma group (*n*=31) ranged between *r*=0.52 and *r*=0.56 (all *p* values<0.001). Correlations between the RCADS and the CPTCI were somewhat larger in the multiple-event trauma sample (*n*=31) than in the single trauma sample (*n*=144) (multiple-event trauma sample, *r*=0.82–0.86; single-event trauma sample, *r*=0.68–0.72; all significant at *p*<0.001). Examination of the correlations between the CPTCI and the KIDSCREEN-10 showed a strong negative association (see Table 2).

**Table 2:** Pearson correlations of the CPTCI, CAPS-CA, and RCADS subscales and the KIDSCREEN-10

Measure	CPTCI-PC	CPTCI-SW	CPTCI total
CPTCI (*N*=502)			
CPTCI-PC	–		
CPTCI-SW	0.77***	–	
CPTCI total	0.95***	0.93***	–
CAPS-CA (*n*=80)			
Cluster B	0.55***	0.50***	0.55***
Cluster C	0.59***	0.61***	0.63***
Cluster D	0.49***	0.50***	0.51***
Total PTSD severity	0.64***	0.63***	0.66***
RCADS (*n*=175)			
Panic disorder	0.59***	0.57***	0.61***
Social phobia	0.57***	0.61***	0.62***
Major depressive disorder	0.73***	0.71***	0.76***
Separation anxiety disorder	0.37***	0.36***	0.38***
General anxiety disorder	0.62***	0.64***	0.66***
Obsessive compulsive disorder	0.59***	0.53***	0.59***
Total internalizing scale	0.72***	0.72***	0.76***
KIDSCREEN-10 (*n*=313)	−0.40***	−0.41***	−0.44***

CPTCI=Child Posttraumatic Cognitions Inventory; CPTCI-PC=permanent and disturbing change subscale of the CPTCI; CPTCI-SW=fragile person in a scary world subscale of the CPTCI; CAPS-CA=Clinician-Administered PTSD Scale for Children and Adolescents; RCADS=Revised Child Anxiety and Depression Scale.

The CAPS-CA and the RCADS were administered in the clinical sample; the KIDSCREEN-10 was administered in the school sample.

*** *p*<0.001.

### Ability of the CPTCI to discriminate between children in the clinical sample and the school sample

We found significantly higher scores on the CPTCI in the clinical sample compared to the school sample: for the CPTCI-PC subscale *t*(500)=−5.73, *p*<0.001; for the CPTCI-SW subscale *t*(1, 500)=−5.78, *p*<0.001; and for the CPTCI total scale *t*(500)=−6.13, *p*<0.001.

### Measurement of treatment effects

In a subsample of 23 children from the clinical sample, we found a strong, significant correlation between CPTCI change scores from pre- to posttreatment and CAPS-CA change scores from pre- to posttreatment *r*=0.54, *p*=0.01. For 108 children in the clinical sample, we also calculated correlations between CPTCI change scores and change scores on the RCADS. These were also significant (all *p* values<0.05) and ranged between *r*=0.24 and 0.46.

### Age and gender differences

Means and standard deviations for the CPTCI total and subscales per age group and for boys and girls separately are displayed in Table 3. There were no significant differences between the age groups. Neither the overall comparison nor the post hoc group comparisons were significant (all *p* values>0.05). Comparisons of the CPTCI scores for boys and girls revealed that girls scored significantly higher on the subscales and the total scale (all *p* values<0.001).

**Table 3:** Means and standard deviations for CPTCI (sub)scales by sample and age group

	CPTCI-PC		CPTCI-SW		CPTCI total	
		
	*M*	*SD*	*M*	*SD*	*M*	*SD*
Boys (*n*=230)	19.53	6.83	19.87	5.80	39.40	11.81
Girls (*n*=271)	22.17	8.36	22.96	7.42	45.13	14.89
8–11 (*n*=155)	22.17	7.31	22.53	5.54	44.70	11.90
12–14 (*n*=104)	20.63	8.02	21.09	6.92	41.72	14.11
15–18 (*n*=243)	20.30	7.94	21.11	7.57	41.41	14.72
School (*n*=318)	19.47	6.92	20.24	6.14	39.71	12.13
Clinic (*n*=184)	23.49	8.55	23.81	7.50	47.30	15.25
Pretreatment (*n*=118)	22.80	9.20	23.23	7.01	46.09	14.32
Posttreatment (*n*=118)	17.39	6.26	18.79	6.42	36.19	12.28

CPTCI=Child Posttraumatic Cognitions Inventory; CPTCI-PC=permanent and disturbing change subscale of the CPTCI; CPTCI-SW=fragile person in a scary world subscale of the CPTCI; clinic=clinical sample; school=school sample.

## Discussion and conclusion

The present validation study showed that the Dutch version of the CPTCI has good psychometric properties. The CFAs indicated that the two-factor model provided a mediocre fit of the data, which was superior to a single-factor model. The fact that the correlation between the two subscales was strong but lower than 0.8 and that both subscales independently of each other correlated (almost) significantly with the CAPS-CA severity score also supports the two-factor model. Inspection of the factor loadings furthermore suggested that the two-factor structure provided a satisfactory fit of the data. Our factor loadings resembled the ones Meiser-Stedman, Dalgleish, et al. (2009) found in their school and their 6-months post-trauma sample. Like in the original version, we found adequate factor loadings for all but the item “Life is not fair” in one of the two samples. Surprisingly, whereas Meiser-Stedman et al. found a low factor loading for this item in the clinical sample, we found the low factor loading in the school sample. However, neither in the total sample nor in the subsamples did the item influence Cronbach's *α* of the sub- or total scale negatively, indicating that it fits the scales. Our Cronbach's *α*'s for the two subscales were comparable to those of the original English version. They were larger for older than for younger children indicating that the CPTCI is more reliable in older age groups.

Further investigation of the validity of the CPTCI showed that the questionnaire correlated positively with measures of PTSD, depression, and anxiety, and negatively with quality of life. In general, correlations between the CPTCI and the CAPS-CA and the CPTCI and the RCADS were strong. Since PTSD is often accompanied by symptoms of depression and anxiety, this finding is not surprising. Also important to consider is that dysfunctional beliefs are not restricted to PTSD but are also present in depression and anxiety disorder (Beck, 2005). However, after controlling for depression and anxiety, the CPTCI still correlated significantly with the total CAPS-CA score. This indicates that the correlation between trauma-related cognitions and PTSD severity is not just an artifact of depression or anxiety. Additional support for the validity of the CPTCI offers the negative correlation with the KIDSCREEN-10. As quality of life increases, trauma-related cognitions decrease. With respect to its ability to discriminate between groups, we found that the CPTCI was able to discriminate between children in the clinical sample and children in the school sample. Children in the clinical sample scored significantly higher than children in the school sample. Furthermore, for children who received treatment we found that a decrease in CPTCI scores was accompanied by a decrease in CAPS-CA scores and decrease in RCADS score. Since trauma-focused therapy has shown not only to reduce PTSD symptoms but also comorbid symptoms (see for an overview Gillies, Taylor, Gray, O'Brien, & D'Abrew, 2013), these findings support the assumption that the CPTCI is able to detect treatment effects. This quality makes it a valuable instrument for treatment outcome studies in children with PTSD since in the DSM-5 trauma-related cognitions are part of the PTSD diagnostic criteria.

Our subgroup analyses of children who were exposed to a single-event trauma and children who were exposed to multiple-event trauma suggest that the CPTCI performs well in both groups. Independently of the sample in which the analyses were performed, the internal consistency was good and correlations with PTSD and anxiety and depression were strong. These findings indicate that the CPTCI is a useful tool not only for children who were exposed to single-event trauma but also for children who were exposed to multiple-event trauma. In our additional subgroup analyses, we found significantly higher scores for girls than for boys on the CPTCI subscales and the total scale. These gender differences match findings from earlier studies. In both child and adult studies, female respondents score higher on the (C)PTCI than male respondents (e.g., Daie-Gabai et al., 2011; Meiser-Stedman, Smith, et al., 2009). This difference has previously been explained by the fact that girls are generally more prone to display internalizing behavior, which is strongly associated with trauma-related cognitions like our results indicate, whereas boys more often display externalizing behavior (e.g., Meiser-Stedman, Smith, et al., 2009; Muris, Van der Pennen, Sigmond, & Mayer, 2008). This explanation is also supported by our results: In the current sample, we found higher scores on the RCADS total internalizing scale for girls than for boys. Consistent with the results from Meiser-Stedman, Smith, et al. (2009), we found no significant differences on the CPTCI scales between the three age groups.

Some limitations should be mentioned. As often is the case in validation studies, our school sample was much larger than our clinical sample. Sample sizes varied also with respect to the administration of the questionnaires and the CAPS-CA. Due to time restrictions, we did not administer the RCADS in the school sample but chose to administer a short, less time consuming quality of life questionnaire. Since the CAPS-CA was only part of the standard test battery in Amsterdam, our sample was restricted to that clinical group. Although our sample was quite heterogeneous with respect to traumatic events that children reported, the group of children who was referred to the centers for child and adolescent trauma as having experienced multiple-event trauma was quite small. Therefore, we were restricted in the analyses that we could perform in this subsample and our results with respect to this group should be interpreted with caution. Additional research of the qualities of the CPTCI in this particular group is needed. Another point that deserves attention is the reliability of the CPTCI in the age group 8–11 years. Although the internal consistency for the CPTCI-PC subscale was acceptable, it was relatively low in comparison to internal consistencies we found in the older age groups. Future studies should look further into the reliability of the CPTCI in young children. Furthermore the factor structure of the CPTCI deserves more attention. Our results generally support the two-factor solution. However, since the model fit was not overly convincing, a different factor structure or item constellation cannot be completely ruled out either. With our validation study we replicated results of previous studies and showed that the translation of the questionnaire has as good psychometric properties as the original English version and can be used without major adaptations. Since the present and former studies mainly focused on the investigation of the construct validity of the CPTCI, investigations of its criterion validity by means of receiver operating characteristic analysis, for example, would be beneficial. Despite these limitations, we conclude that the Dutch CPTCI is a reliable and, with respect to construct validity, valid instrument. This study furthermore demonstrated that the CPTCI can be used to measure trauma-related cognitions in children who were exposed to different kinds of traumatic events and that its qualities are not limited to a specific trauma type.

## Supplementary Material

The Dutch version of the Child Posttraumatic Cognitions Inventory: validation in a clinical sample and a school sample
