# Supplementary material for: The Dutch version of the Child Posttraumatic Cognitions Inventory: validation in a clinical sample and a school sample
Source: Eur J Psychotraumatol. 2015 Feb 23;6:10.3402/ejpt.v6.26362. doi: 10.3402/ejpt.v6.26362 (PMC4344547; doi:10.3402/ejpt.v6.26362)
Supplement: The Dutch version of the Child Posttraumatic Cognitions Inventory: validation in a clinical sample and a school sample [file EJPT-6-26362-s001.pdf]

Title: La versión holandesa del Inventario de Cogniciones Postraumáticas Infantiles: Validación en una muestra clínica y una muestra de la escuela

Julia Diehle, Carlijn de Roos, Richard Meiser-Stedman, Frits Boer, Ramón J.L. Lindauer

Abstract
Antecedentes: Con la inclusión de las cogniciones relacionadas con el trauma en los criterios del DSM-5 para el trastorno de estrés postraumático, la valoración de estas cogniciones se ha convertido en algo esencial. Por lo tanto, está justificado el uso de herramientas válidas para la evaluación de estas cogniciones.
Objetivo: El presente estudio pretende a validar la versión holandesa del inventario de cogniciones postraumáticas infantiles (CPTCI).
Método: Incluimos en nuestro estudio a niños de 8 a 19 años y evaluamos la estructura factorial, la fiabilidad y la validez del CPTCI en una muestra clínica (n = 184) y una muestra de la escuela (n = 318).
Resultados: Nuestros resultados apoyan la estructura de dos factores del CPTCI y muestran una buena consistencia interna para el total de la escala y las dos subescalas. Se encontraron correlaciones positivas significativas entre el CPTCI y las medidas de trastorno de estrés postraumático, depresión y trastorno de ansiedad. El CPTCI se correlacionó negativamente con una medida de calidad de vida. Además encontramos puntuaciones significativamente más altas en la muestra clínica que en la muestra de la escuela. Para los niños que recibieron tratamiento, se encontró que la disminución en las puntuaciones del CPTCI iba acompañada por una disminución de los síntomas de estrés postraumático y problemas co-mórbidos, lo que indica que el CPTCI es capaz de detectar los efectos del tratamiento.
Conclusión: En general, nuestros resultados sugieren que el CPTCI holandés es un instrumento fiable y válido.

Keywords: Trastorno de estrés postraumático; niños; cogniciones; fiabilidad; validez

Name of translator: Miriam Ramos Morrison

Citation: European Journal of Psychotraumatology 2015, 6: 26632 - http://dx.doi.org/10.3402/ejpt.v6.26632
